# Longitudinal lung function in childhood cancer survivors after hematopoietic stem cell transplantation

**DOI:** 10.1038/s41409-021-01509-1

**Published:** 2021-11-08

**Authors:** Maria Otth, Sophie Yammine, Jakob Usemann, Philipp Latzin, Luzius Mader, Ben Spycher, Tayfun Güngör, Katrin Scheinemann, Claudia E. Kuehni, for the Swiss Pediatric Oncology Group (SPOG), M. Ansari, M. Beck Popovic, J. P. Bourquin, P. Brazzola, J. Greiner, J. Rössler, F. Schilling, K. Scheinemann, N. von der Weid

**Affiliations:** 1 Childhood Cancer Research Group, Institute of Social and Preventive Medicine (ISPM), University of Bern, Bern, Switzerland; 2 Division of Oncology- Hematology, Department of Pediatrics, Kantonsspital Aarau, Aarau, Switzerland; 3 Graduate School for Cellular and Biomedical Sciences, University of Bern, Bern, Switzerland; 4 Department of Oncology, Hematology, Immunology, Stem Cell Transplantation and Somatic Gene Therapy, University Children’s Hospital Zurich – Eleonore Foundation, Zurich, Switzerland; 5 Division of Respiratory Medicine, Department of Pediatrics, Inselspital, University Hospital, University of Bern, Bern, Switzerland; 6 Division of Respiratory Medicine, Department of Pediatrics, University Children’s Hospital Zurich-Eleonore Foundation, Zurich, Switzerland; 7 University Children’s Hospital Basel (UKBB), Basel, Switzerland; 8 University of Basel, Basel, Switzerland; 9 Department of Pediatrics, McMaster University Hamilton, Hamilton, ON Canada; 10 Division of Hematology/Oncology, Department of Pediatrics, Inselspital, University Hospital, University of Bern, Bern, Switzerland; 11 CANSEARCH Research Laboratory, Medical Faculty, University of Geneva, Geneva, Switzerland; 12 Department of Women, Children and Adolescents, Division of Pediatric Oncology and Hematology, Geneva University Hospital, Geneva, Switzerland; 13 Pediatric Hematology-Oncology Unit, Lausanne University Hospital, Centre Hospitalier Universitaire Vaudois, Lausanne, Switzerland; 14 Pediatria Bellinzona, Ospedale Regionale di Bellinzona e Valli, Bellinzona, Switzerland; 15 Division of Hematology and Oncology, Children’s Hospital of Eastern Switzerland, St. Gallen, Switzerland; 16 Division of Pediatric Hematology/Oncology, Department of Pediatrics, Inselspital, Bern University Hospital, University of Bern, Bern, Switzerland; 17 Division of Oncology/ Hematology, Department of Pediatrics, Kantonsspital Luzern, Lucerne, Switzerland; 18 Division of Hematology and Oncology, University Children’s Hospital Basel and University of Basel, Basel, Switzerland

**Keywords:** Cancer, Paediatrics, Epidemiology

## Abstract

Longitudinal data on pulmonary function after pediatric allogeneic or autologous hematopoietic stem cell transplantation (HSCT) are rare. We examined pulmonary function and associated risk factors in 5-year childhood cancer survivors (CCSs) longitudinally. We included 74 CCSs diagnosed between 1976 and 2010, treated with HSCT, and with at least two pulmonary function tests performed during follow-up. Median follow-up was 9 years (range 6–13). We described pulmonary function as z-scores for lung volumes (forced vital capacity [FVC], residual volume [RV], total lung capacity [TLC]), flows (forced expiratory volume in 1 s [FEV1], maximal mid-expiratory flow [MMEF]), and diffusion capacity for carbon monoxide (DLCO) and assessed associations with potential risk factors using multivariable regression analysis. The median z-scores for FEV1, FVC, and TLC were below the expected throughout the follow-up period. This was not the case for RV, MMEF and DLCO. Female gender, radiotherapy to the chest, and relapse were associated with lower z-scores of FEV1, FVC, MMEF, RV or DLCO. Childhood cancer survivors after HSCT are at risk of pulmonary dysfunction. The complex and multifactorial etiology of pulmonary dysfunction emphasizes the need for longitudinal prospective studies to better characterize the course and causes of pulmonary function impairment in CCSs.

## Introduction

Childhood cancer survivors (CCSs) treated with hematopoietic stem cell transplantation (HSCT) are at increased risk of pulmonary dysfunction, which reflects different structural and functional damage to the lung [1–4]. This can result from oxidative stress induced by lung-toxic chemotherapeutics (busulfan, bleomycin, carmustine and lomustine), free radical formation during radiotherapy, or transplant-specific pulmonary complications, such as idiopathic pulmonary syndrome or bronchiolitis obliterans [5–8]. Because the lung has a large functional reserve, it can take years to decades until pulmonary dysfunction manifests with symptoms. Pulmonary function testing (PFT) might allow to detect pulmonary dysfunction during the pre-symptomatic period. Spirometry and body plethysmography measuring lung volumes and flow, are widely available but supposedly less sensitive than the diffusion capacity for carbon monoxide (DLCO) [9, 10].

Literature on longitudinal course of pulmonary function in CCSs after HSCT is sparse and previous studies had only short follow-up periods of up to 6.8 years [3, 4, 11, 12]. With this population-based retrospective cohort study we aimed to close this knowledge gap by describing pulmonary function trajectories up to 15 years from cancer diagnosis and to investigate predictors of pulmonary dysfunction in a cohort of long-term CCSs treated with HSCT.

## Methods

### Study population and design

The study population consisted of participants of the Swiss Childhood Cancer Survivor Study (SCCSS), a questionnaire-based, national cohort study of all children and adolescents registered in the Swiss Childhood Cancer Registry (SCCR), who had survived ≥5 years [13, 14]. We included SCCSS participants, who had been treated in a Swiss pediatric oncology clinic between 1976 and 2010, had undergone autologous or allogeneic HSCT, and had at least two pulmonary function tests (PFTs) performed within 15 years after the cancer diagnosis (Supplementary Fig. S1). We collected information on treatment and PFT results from medical records at the clinics where the patients had been treated or had received HSCT. The Ethics Committee of the Canton of Bern approved the SCCR and SCCSS (KEK-BE: 166/2014). The SCCSS is registered at ClinicalTrials.gov (identifier: NCT03297034).

### Pulmonary function tests

We extracted the following PFT parameters from medical records: forced expiratory volume in first second (FEV1), forced vital capacity (FVC), and maximal mid-expiratory flow (MMEF) from spirometry, residual volume (RV) and total lung capacity (TLC) from body plethysmography, and DLCO corrected for hemoglobin if available. We divided DLCO expressed as [cmH_2_O/L/sec] by 2.98 to convert it into [mmol/min/kPa] [15]. We used the Global Lung function Initiative equations (GLI 2012) to convert FEV1, FVC, and DLCO into age-, height- and sex-standardized z-scores. For MMEF, TLC, and RV, where no GLI reference values are available, we used the reference equations by Zapletal et al. for children (4–17 years) and the European Community of Coal and Steel (ECCS) equations for adults (≥18 years) [16–18]. We defined lower limits of normality as z-scores of less than −1.645 [19]. We checked z-scores for outliers and corrected data entry errors if necessary. We excluded PFT results if poor cooperation, cough or cold were noted in the records. Two authors independently assessed PFT quality by evaluating the flow-volume curve and loop according to official criteria [20, 21]. We excluded 23 tests of 8 CCSs that were done ≥15 years from cancer diagnosis (mean 23 years; range 15–34), as such long-term data were available for few only. Including those might have led to selection bias.

### Treatment characteristics

We extracted information on all chemotherapeutics classified as lung-toxic in long-term follow-up guidelines (bleomycin, busulfan, carmustine, and lomustine) from medical records and calculated cumulative doses [22–24]. We converted busulfan administered orally to intravenously by multiplying it by 0.8 [25]. We additionally extracted information on thoracic radiotherapy and surgery [22]. For HSCT we collected information on source of transplant, stem cell donor, and graft versus host disease (GvHD) and categorized GvHD into acute and chronic according to medical records.

### Statistical analyses

We used medians and interquartile ranges (IQR), numbers and proportions to characterize the study population and the PFT results. We compared results from the first and last PFT of each CCS using Wilcoxon rank-sum test. This was not done for DLCO, which was missing in >50% of first or last tests. We plotted the longitudinal trajectories of pulmonary function parameters over time for each CCS, and the median of the cohort. In a sub-cohort we analyzed FEV1 and FVC z-scores before HSCT (baseline), <2 years, 3–4 years, and ≥5 years from HSCT. If CCSs had more than one PFT within one of these time periods, we took the mean of these tests. To assess predictors of the longitudinal course of pulmonary function parameters, we used mixed effects multivariable linear regression analysis including a random intercept and random slope for time since diagnosis, to account for repeated measurements within patients. We included gender, type of HSCT, thoracic radiotherapy, lung-toxic chemotherapy, relapse, and decade of diagnosis in all models. Additionally we tested possible interactions between these variables and time since diagnosis. We included interaction terms in the final model if the *p* value was <0.05. The model allowed for correlation of residuals within survivors using an exponential autocorrelation function. Male patients treated with autologous HSCT, diagnosed in 1980–1990, and not exposed to any of the other risk factors were modeled as a reference (Supplementary Explanation E1). In a sensitivity analysis, we restricted the follow-up period to the first five years after diagnosis, to assess whether risk factors had a greater influence on changes in pulmonary function in the first five years compared to the entire observation period. We used the statistical software Stata (StataCorp LLC, Version 16).

## Results

### Patient characteristics

Among 142 SCCSS responders treated with HSCT, we found at least two PFTs of good quality in the medical records of 74 CCS (Supplementary Fig. 1). The median age at diagnosis was 7.4 years (IQR 3.5–12.2). The median time between diagnosis and HSCT was 0.8 years (IQR 0.5–2.6). The most frequent diagnosis was leukemia (69%). Busulfan was the most frequently used lung-toxic treatment. Seventy percent of CCSs had received thoracic radiotherapy,14% thoracic surgery. Most CCSs were transplanted allogeneic (68%) (Table 1). Additional information on cancer diagnosis, transplant source, and GvHD is available in Supplementary Table S1.

**Table 1:** Characteristics of the study population of 5-year childhood cancer survivors treated with hematopoietic stem cell transplantation in Switzerland, *N* = 74.

Sociodemographic and lifestyle characteristics	*n* (%)
Sex, male	43 (58)
Ethnicity, white	72 (97)
Age at first lung function test, median years (IQR)	9.9 (7.9–14.0)
Age at last lung function test, median years (IQR)	16.2 (14.2–20.0)
Smoking status^a^ Active smoking Former active smoking Passive smoking Never active smoking	4 (5) 5 (7) 32 (43) 33 (45)
**Clinical characteristics**	
Age at diagnosis, median years (IQR)	7.4 (3.5–12.2)
Time between diagnosis and HSCT, median years (IQR)	0.8 (0.5–2.6)
Follow-up, median years (IQR)^b^	9.4 (6.1–12.3)
Era of diagnosis 1980–1990 1991–2000 2001–2010	8 (11) 24 (32) 42 (57)
Cancer diagnosis according to ICCC-3 I: Leukemia II: Lymphoma Other^c^	51 (69) 12 (16) 11 (15)
Relapse	41 (55)
**Treatment characteristics**	
Lung-toxic chemotherapeutics, dose, mg/m^2^ (IQR)	
Busulfan (*n* = 25)	422 (324–470)
Carmustine (*n* = 5)	300 (300–300)
Lomustine (*n* = 1)	190
Bleomycin (*n* = 4)	41 (30–46)
Radiotherapy involving the thorax^d^	52 (70)
Conditioning containing TBI	39 (53)
Thoracic surgery^e^	10 (14)
**Transplant characteristics**	
Stem cell donor	
Autologous	24 (32)
Allogeneic	50 (68)
HLA identical sibling / HLA matched (un-)related donor	29 (58)
HLA mismatch (un-)related / haploidentical	11 (22)
Missing	10 (20)

^a^For categorization of smoking status see Supplementary Material.

^b^Time between diagnosis and last pulmonary function test.

^c^Other tumors include: tumor of the central nervous system (*n* = 1), retinoblastoma (*n* = 1), malignant bone tumor (*n* = 5), soft tissue sarcoma (*n* = 3), malignant germ cell tumor (*n* = 1*)*, neuroblastoma (*n* = 1).

^d^Thoracic radiation fields according to COG guidelines, Version 4.0, Oct 2018, including radiation to the chest, whole lung, mediastinum, (mini-)mantle field, TBI and additionally upper abdomen, thoracic spine, and craniospinal irradiation.

^e^Thoracic surgery according to COG guidelines, Version 4.0, Oct 2018, including thoracotomy, chest wall surgery, rib resection, lobectomy, pulmonary metastasectomy and wedge resection.

### Pulmonary function

Of the 74 CCSs we retrieved 411 good quality PFTs, on average 5 tests per survivor (range 2–12). The median time from diagnosis to the first PFT was 3 years (IQR 1–5) and 9 years (IQR 6–12) to the last PFT. Because not all six outcome parameters (FEV1, FVC, FEV1/FVC, MMEF, TLC, and RV) had been measured in the first and the last test, we analyzed changes between the two tests for FEV1 (72 CCSs), FVC (66 CCSs), MMEF (42 CCSs), TLC (58 CCSs), and RV (55 CCSs.) For the longitudinal analysis, FEV1 was available from 407 PFTs, TLC from 390, and DLCO from 185. We could analyze 147 tests of 25 CCS which had at least one test performed before HSCT.

**Fig. 1:**
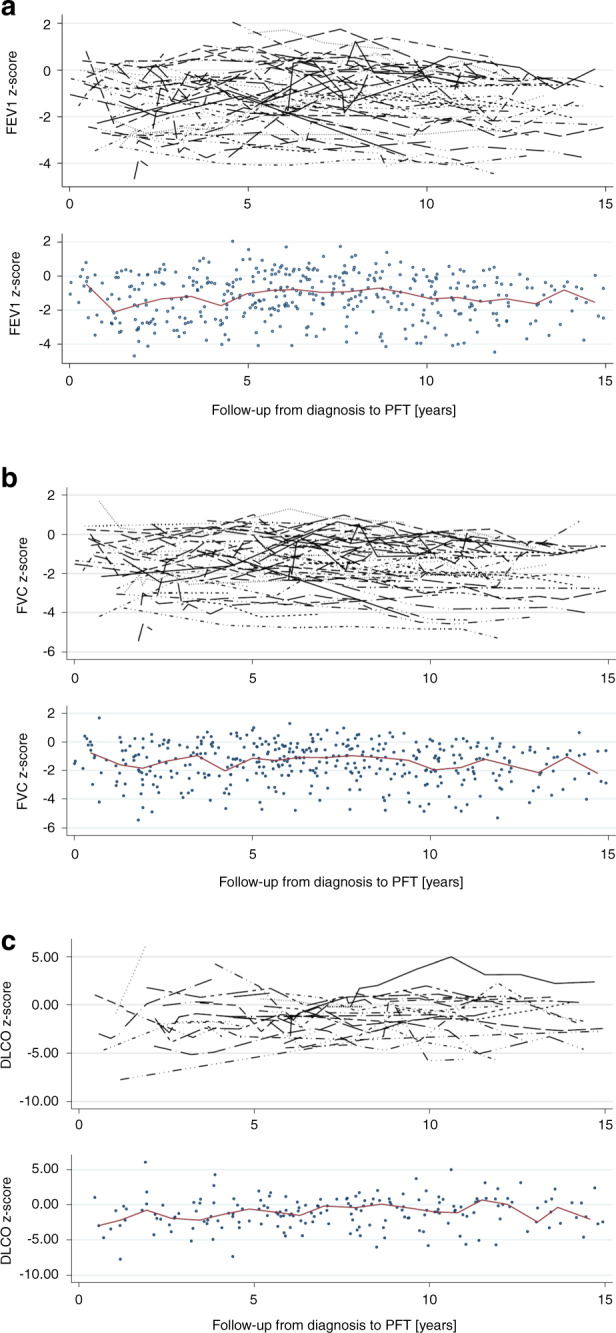
Longitudinal pulmonary function trajectories in childhood cancer survivors following HSCT. Longitudinal trajectories of (**a**) FEV1 z-score, (**b**) FVC z-score, and (**c**) DLCO z-score over time, upper part showing the trajectory of each patient, lower part showing the median of all observations.

**Fig. 2:**
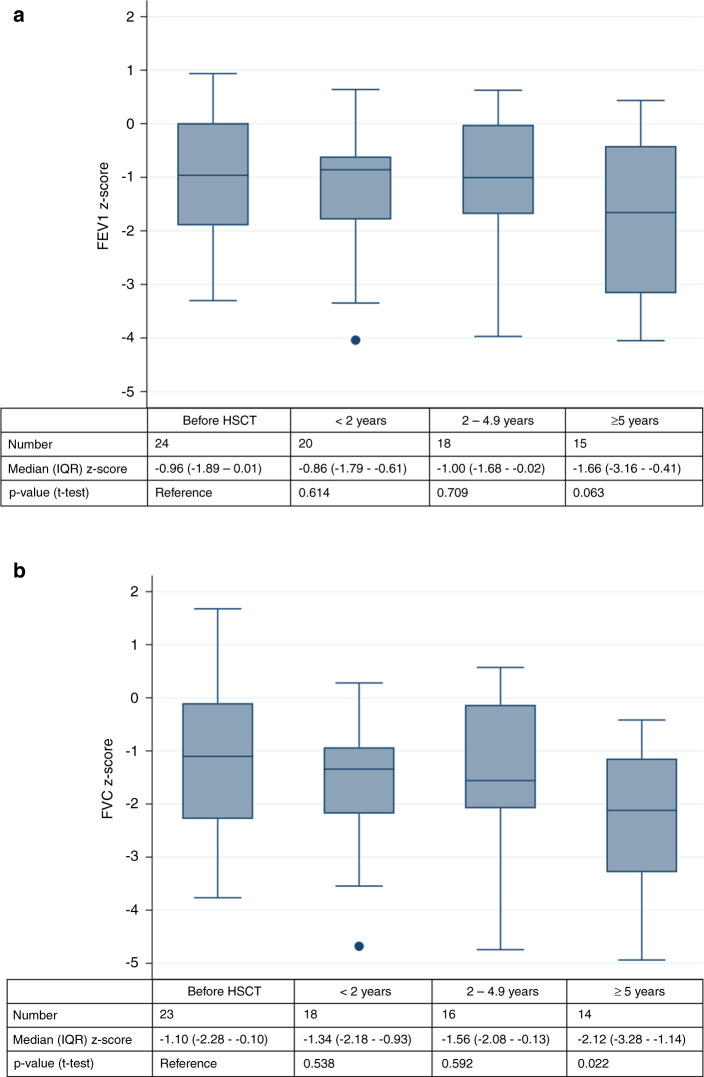
Median FEV1 and FVC z-scores in 24 childhood cancer survivors with pulmonary function testing before HSCT. **a** Course of FEV1, **b** Course of FVC. T-test comparing before HSCT with follow-up categories.

Half of the CCSs (51%) had at least one abnormal parameter in their last test (Table 2). Only the FEV1/FVC ratio decreased significantly between the first and the last test. The median z-scores of FEV1, FVC, and MMEF tended to be lower in the last test, TLC tended to increase in the last test. Also RV was slightly above predicted at both time points (Table 2). Graphically, the median z-score of each pulmonary function parameter remained below the expected for FEV1, FVC, and TLC, and undulated around the expected for MMEF, DLCO and RV (Fig. 1, Supplementary Fig. S2). We observed a large inter-individual variability in the longitudinal trajectories of all lung function parameters. In some CCS parameters deteriorated or improved over time in others they showed a variable course. Of the 25 CCSs who had a baseline PFT, FEV1 was available for 24 CCSs and FVC for 23. The median FEV1 z-score deteriorated from −0.96 at baseline (IQR −1.89–0.01) to −1.66 (IQR −3.16–−0.41) in tests performed ≥5 years from HSCT (Fig. 2a). We also observed a significant decrease from baseline to ≥5 years from HSCT for FVC (Fig. 2b). Supplementary Fig. S3 shows the changes of FEV1 and FVC z-scores for all CCS who had a baseline test before HSTC. The reasons why only some CCS were followed-up for more than 5 years was not evident from the available data.

**Table 2:** Comparison of first and last available pulmonary function test in transplanted childhood cancer survivors; *N* = 74.

	FIRST test		LAST test			
	*n*^a^ (%)	Median (IQR)	*n*^a^ (%)	Median (IQR)	Mean difference	*p* value^b^
**Follow-up**^**c**^ **[years]**		3.04 (1.23–5.38)		9.33 (6.08–12.27)		
**FEV1 z-score**						
Whole cohort	72	−0.76 (−2.12–0.08)	72	–1.01 (−2.19–−0.55)	−0.32 (−1.09–0.35)	0.115
Normal	48 (67)		47 (65)			
Reduced	24 (33)		25 (35)			
**FVC z-score**						
Whole cohort	66	−0.87 (−2.18–−0.22)	66	−1.16 (−2.21–−0.61)	−0.31 (−0.96–0.35)	0.182
Normal	45 (68)		41 (62)			
Reduced	21 (32)		25 (38)			
**FEV1/FVC**						
Whole cohort (ratio)	66	0.93 (0.89–0.97)	66	0.91 (0.87–0.94)	−0.02 (−0.05–0.01)	**0.031**
Ratio ≥0.7	65 (99)		65 (99)			
Ratio <0.7	1 (1)		1 (1)			
**MMEF**						
Whole cohort	42	0.11 (−1.11–0.94)	42	−0.07 (−0.98–0.35)	−0.14 (−0.85–0.85)	0.421
Normal	35 (83)		39 (93)			
Reduced	7 (17)		3 (7)			
**TLC z-score**						
Whole cohort	58	−1.26 (−2.55–−0.22)	58	−0.81 (−2.14–0.23)	0.15 (−0.97–1.44)	0.309
Normal	33 (58)		39 (68)			
Reduced	25 (42)		19 (32)			
**RV z-score**						
Whole cohort	55	0.55 (−1.22 – 2.05)	55	0.31 (−0.51–1.32)	−0.43 (−1.44–0.87)	0.521
Normal	49 (89)		50 (91)			
Reduced	6 (11)		5 (9)			
**At least one abnormal test**	44 (59)		38 (51)			

Normal: measured value ≥ −1.645 z-score.

Reduced: measured value < −1.645 z-score.

^a^Total number of CCSs differ between lung function parameters as not all survivors had all parameters performed at first and last test.

^b^Wilcoxon rank sum test comparing median z-score between first and last test.

^c^Time between diagnosis and pulmonary function test.

### Risk factors for decreased pulmonary function

Being female, thoracic radiotherapy, and relapse were associated with lower lung function. Females had lower intercepts for FEV1 and MMEF, but no differences in the slopes for any parameter (Table 3). Treatment with radiotherapy was associated with lower intercepts for FEV1 (−1.31 z-scores; 95% CI −2.06–−0.56) and FVC (−1.47 z-scores 95% CI −2.21–−0.74), but not with slopes. Relapse was associated with higher intercepts for TLC, but contributed to a decrease over time of TLC and RV. Children treated more recently had lower intercepts for DLCO. We observed no effect of transplantation type or treatment with lung-toxic chemotherapy on the intercept or slope of any parameter (Table 3). The results remained similar in the sensitivity analysis restricted to the first five years after diagnosis (Supplementary Tables S2–S7).

**Table 3:** Effects of risk factors on longitudinal changes in FEV1, FVC, MMEF, TLC, RV, and DLCO z-scores in childhood cancer survivors after hematopoietic stem cell transplantation with repeated pulmonary function tests available.

	Coefficient^b^	*p*^c^	95% Conf. Interval	
**FEV1 z-score** (*n* = 407 PFTs, 74 CCSs)				
**Final model**^a^				
Intercept	0.499	0.69	−0.921	1.919
Gender (ref. male)	−0.664	**0.013**	−1.187	−0.140
Type of HSCT (ref. autologous)	0.481	0.113	−0.113	1.076
Radiotherapy to lung (ref. no)	−1.306	**0.001**	−2.055	−0.558
Lung-toxic chemotherapy (ref. no)	−0.559	0.123	−1.270	0.152
Relapse (ref. no)	0.395	0.154	−0.148	0.937
Decade of diagnosis (ref. 1980–1990)				
1991–2000	−0.667	0.183	−1.647	0.314
2001–2010	−0.197	0.678	−1.131	0.736
Time since diagnosis [decrease per year]	−0.061	**<0.001**	−0.094	−0.027
**FVC z-score** (*n* = 395 PTFs, 73 CCSs)				
**Final model**^a^				
Intercept	0.147	0.837	−1.252	1.546
Gender (ref. male)	−0.387	0.152	−0.916	0.143
Type of HSCT (ref. autologous)	0.533	0.077	−0.058	1.123
Radiotherapy to lung (ref. no)	−1.473	**<0.001**	−2.207	−0.739
Lung-toxic chemotherapy (ref. no)	−0.647	0.069	−1.347	0.051
Relapse (ref. no)	0.237	0.395	−0.309	0.783
Decade of diagnosis (ref. 1980–1990)				
1991–2000	−0.312	0.531	−1.287	0.663
2001–2010	−0.083	0.861	−1.008	0.843
Time since diagnosis [decrease per year]	−0.058	**0.003**	−0.097	-0.019
**MMEF z-score** (*n* = 268 PTFs, 61 CCSs)				
**Final model**^a^				
Intercept	1.340	0.146	−0.469	3.149
Gender (ref. male)	−0.887	**0.008**	−1.538	−0.237
Type of HSCT (ref. autologous)	0.402	0.328	−0.404	1.208
Radiotherapy to lung (ref. no)	−0.664	0.156	−1.583	0.253
Lung-toxic chemotherapy (ref. no)	−0.494	0.275	−1.383	0.394
Relapse (ref. no)	0.374	0.252	−0.266	1.016
Decade of diagnosis (ref. 1980–1990)				
1991–2000	−1.142	0.059	−2.327	0.042
2001–2010	−0.982	0.106	−2.172	0.208
Time since diagnosis [decrease per year]	−0.003	0.918	−0.058	0.052
**TLC z-score** (*n* = 390 PFT, 74 CCSs)				
**Final model: time interaction for type of HSCT and relapse**				
Intercept	−1.584	0.251	−4.293	1.124
Gender (ref. male)	0.729	0.134	−0.224	1.682
Type of HSCT (ref. autologous)	−0.510	0.479	−1.923	0.903
Radiotherapy to lung (ref. no)	−0.717	0.292	−2.051	0.616
Lung-toxic chemotherapy (ref. no)	−0.587	0.364	−1.855	0.681
Relapse (ref. no)	1.704	**0.015**	0.333	3.075
Decade of diagnosis (ref. 1980–1990)				
1991–2000	−1.052	0.241	−2.814	0.711
2001–2010	−0.204	0.811	−1.874	1.465
Time since diagnosis (continuous per year)	0.103	0.236	−0.067	0.272
Interaction Type of HSCT (ref. autolog.)	0.123	0.136	−0.038	0.284
Interaction relapse (ref. no)	−0.258	**0.001**	−0.414	−0.103
**RV z-score** (*n* = 382 PFTs, 74 CCSs)				
**Final model**^a^				
Intercept	−0.309	0.764	−2.326	1.707
Gender (ref. male)	0.036	0.918	−0.650	0.722
Type of HSCT (ref. autologous)	−0.155	0.692	−0.923	0.612
Radiotherapy to lung (ref. no)	0.663	0.181	−0.307	1.634
Lung-toxic chemotherapy (ref. no)	−0.298	0.518	−1.202	0.606
Relapse (ref. no)	1.085	0.100	−0.208	2.378
Decade of diagnosis (ref. 1980–1990)				
1991–2000	−0.785	0.226	−2.055	0.485
2001–2010	−0.127	0.838	−1.346	1.902
Time since diagnosis (continuous per year)	0.108	0.095	−0.019	0.234
Interaction relapse (ref. no)	−0.231	**0.010**	−0.405	−0.055
**DLCO z-score** (*n* = 185 PFT, 46 CCSs)				
**Final model**^a^				
Intercept	1.948	0.192	−0.977	4.872
Gender (ref. male)	−0.514	0.341	−1.575	0.546
Type of HSCT (ref. autologous)	0.498	0.381	−0.616	1.613
Radiotherapy to lung (ref. no)	−1.279	0.093	−2.773	0.213
Lung-toxic chemotherapy (ref. no)	−0.707	0.296	−2.033	0.619
Relapse (ref. no)	0.138	0.809	−0.986	1.263
Decade of diagnosis (ref. 1980–1990)				
1991–2000	−2.465	**0.004**	-4.151	−0.780
2001–2010	−2.447	**0.004**	-4.111	−0.784
Time since diagnosis [decrease per year]	0.015	0.748	-0.079	0.111

Significant risk factors are marked in bold.

*CCSs* childhood cancer survivors, *HSCT* hematopoietic stem cell transplantation, *PFT* pulmonary function test result.

^a^If not additionally specified, the final model did not include the interaction with risk factors and time since diagnosis. The interaction was only kept in the final model if risk factors were significant (*p* < 0.05) at separate levels. Additional information are available in the supplement.

^b^Coefficients represent mean z-score difference compared to a male reference patient treated with autologous HSCT between 1980 and 1990, with no radiotherapy to the chest, no lung toxic chemotherapy, and no relapse.

^c^*p* value from Wald test.

## Discussion

In this study of 74 CCSs followed-up to 15 years after HSCT, 51% had at least one abnormal pulmonary function parameter. Median z-scores for FEV1, FVC, and TLC were below expected throughout the entire observation period, with a large variability between CCSs. Median FEV1 and FVC z-scores were already below expected in CCS with baseline testing and further declined with elapsing time. Female gender, radiotherapy, and relapse were associated with reductions in at least one pulmonary function parameter.

Our results, with half of CCSs having at least one reduced pulmonary function parameter, are comparable to other studies (38–77%), with a follow-up of at least 5 years [1–3, 26–28]. Differences in the study populations and in reporting of results may explain the variability between studies. Some studies included CCSs transplanted autologous [28], allogeneic [2, 3, 27], or both [1, 26]. The use of different reference equations complicated the comparison. The population analyzed by Cerveri et al. was most similar to ours. They found that 38% of CCSs had at least one abnormal pulmonary function parameter, defined as z-score < −1.645, 5 years from HSCT, among whom 23% had reduced FVC z-score, 10% reduced FEV1, and none had an abnormal FEV1/FVC [26]. The cohort by Cerveri et al. was similar to our cohort for exposure to radiotherapy (75% vs. 70%) and treatment with busulfan or carmustine, but our cohort was diagnosed more recently (1976–2010 vs. 1986–1994).

Our trajectories of FEV1, FVC, and DLCO are comparable to results from Griese et al., who plotted PFT results of 83 CCSs over 14 defined time points from baseline to 10 years after HSCT [12]. Similar to our study, none of the lung function parameters showed a clear decline over time. However, they found an initial decrease, subsequent improvement, followed by an undulating course. Griese et al. did not analyze MMEF, RV, and TLC. Except for RV and MMEF, all graphical trajectories started at z-scores below expected in our cohort. The use of different reference equations may have contributed to this relatively better start of MMEF and RV. Poor general condition, pulmonary involvement or acute toxicities may have contributed to the negative z-scores of FEV1, FVC, TLC, and DLCO shortly after diagnosis in our cohort. Also the reference equations used may not perfectly fit the Swiss population. While validation of GLI 2021 equations showed satisfactory fitting in some populations [29], there was an underestimation of FEV1/FVC in others [30]. As MMEF is correlated with FEV1 and FVC, we would have expected similar courses [31]. In contrast to FEV1 and FVC, MMEF was normal in most first tests and remained rather stable during follow-up. This makes relevant peripheral airway obstruction rather unlikely.

Four other studies had assessed changes in pulmonary function from baseline, to defined time points thereafter [2, 3, 11, 32]. In our CCSs with baseline testing, median FEV1 and FVC z-scores continuously decreased from a negative baseline value to each of the three follow-up points. In the other studies, the lowest z-scores for both parameters were measured between 3 months and 1 year after HSCT, improved thereafter, followed by a further decrease [3, 11, 32]. The use of broader time categories since HSCT in our study may have potentially masked transient improvement. All studies found that pulmonary function parameters were reduced before HSCT already, probably due to damage by previous treatments or underlying conditions.

Inaba et al. used a mixed effects linear regression analysis with a random slope to identify risk factors [2]. They analyzed 660 baseline and follow-up PFTs of 89 CCSs treated with allogeneic HSCT. Females had significantly steeper slopes for RV, FVC, and TLC in the cohort by Inaba et al., but gender was not related to changes in the slope in our cohort. Exposure to radiotherapy or chemotherapy were not associated with changes in the slope of any parameter in both studies. Inaba et al. included only survivors of hematological malignancies. CCSs of both cohorts were therefore exposed to different treatment combinations and chemotherapeutics, which might contribute to the differences in identified risk factors between the studies. This combined toxicity makes it also difficult to assess the effect of single risk factors. However, the longitudinal trajectories of pulmonary function parameters suggest that CCSs exposed to pulmonary toxic treatment modalities, especially radiotherapy, might benefit from regular screening.

### Strengths and limitations

The strengths of this study are the national design, the large sample size of CCSs with several PFTs, the high-quality data on diagnosis and treatment, and the long follow-up period. The use of z-scores, verification of data entry by a second person, rigorous assessment of pulmonary function quality, control for outliers, and the exclusion of PFTs with poor quality are additional strengths.

The retrospective design with a long observation period may have affected the data availability and quality. We did not know why PFTs have been performed, and not all parameters have been assessed each time. PFTs have been performed in different laboratories with changes in equipment and testing procedures. We assume that the tests have been performed according to standard recommendations [33–35]. Only 71% of eligible CCSs treated with HSCT participated in the SCCSS, and for only half of them (*n* = 74, 37% of the total population) we found at least two pulmonary function tests. We do not know if this has biased our findings. While we found little evidence that response bias affects prevalence estimates in the SCCSS [36], it is possible that CCSs with pulmonary symptoms received more PFTs than asymptomatic CCSs, so that our results might overestimate the burden of pulmonary dysfunction. Not all CCSs with available baseline testing had at least one PFT result at the last follow-up point, which might have introduced attrition bias. However, we found only little differences between the groups (Supplementary Table S8). The cohort is heterogeneous in terms of relapsed disease and exposure to treatment modalities. Therefore, we were unable to understand the precise pathophysiology for the complex and heterogeneous lung function trajectories. In addition, we had no information on pulmonary symptoms, diseases or other co-morbidities prior to the cancer diagnosis other than what was recorded in the hospital records. However, severe lung disease leading to pulmonary restriction is rare in children and we expect that this would have been documented as a secondary diagnosis.

In conclusion, our results confirm that CCSs after HSCT are at high risk of pulmonary dysfunction, but also highlight the complexity and multifactorial etiology of pulmonary problems. This suggests that pulmonary function testing before HSCT is essential to have an individual baseline and that pulmonary long-term follow-up care of CCSs after HSCT including preventive and supportive measures is needed.

## Supplementary information

Online Supplement
