# Supplementary material for: Longitudinal lung function in childhood cancer survivors after hematopoietic stem cell transplantation
Source: Bone Marrow Transplant. 2021 Nov 8;57(2):207–14. doi: 10.1038/s41409-021-01509-1 (PMC8821009; doi:10.1038/s41409-021-01509-1)
Supplement: Supplementary file 1 — Online Supplement [file 41409_2021_1509_MOESM1_ESM.docx]

**Longitudinal Lung Function in Childhood Cancer Survivors after Hematopoietic Stem Cell Transplantation**

Maria Otth; Sophie Yammine, Jakob Usemann; Philipp Latzin, Luzius Mader, Ben Spycher, Tayfun Güngör, Katrin Scheinemann, Claudia E. Kuehni

**Supplementary Tables and Figures**

**SUPPLEMENTAL EXPLANATION E1:** Illustration of the multivariable linear regression analysis with random intercept and random slope. The intercept and slope of the reference person differs for every pulmonary function parameter.

The **intercept** represents time zero, corresponding to the lung function, expressed as z-score, at time of cancer diagnosis. The **slope** describes the change of the lung function outcome parameter over time.

| **Example 1:** Calculation of **FEV1** for a woman treated with allogeneic HSCT, exposed to radiotherapy and lung toxic chemotherapy , and diagnosed 2005 | | |
| --- | --- | --- |
| The **reference person** in this model is a male patient treated with autologous HSCT, diagnosed between 1980 and 1990, and not exposed to any other risk factor | 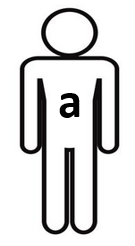  1980 - 1990 | Intercept: 0.499  Slope: -0.061 |
| **Intercept** for female gender: -0.664  **Intercept**  for allogeneic HSCT: 0.481  **Intercept** for radiotherapy yes: -1.306  **Intercept** for chemotherapy yes: -0.559  **Intercept** for decade: -0.197 | 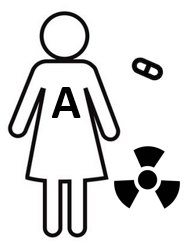  2001 - 2010 | Intercept: -1.746  (= 0.499-0.664+0.481  -1.306-0.559-0.197)  Slope: -0.061 |

| **Example 2:** Calculation of **TLC** for a male patient treated with autologous HSCT, exposed to lung toxic chemotherapy, who suffered from relapse and was diagnosed 1999 | | |
| --- | --- | --- |
| The **reference person** in this model is a male patients treated with autologous HSCT, diagnosed between 1980 and 1990, and not exposed to any of the other risk factors | 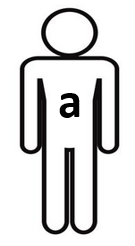  1980 - 1990 | Intercept: -1.584  Slope: 0.103 |
| **Intercept** for chemotherapy yes: -0.587  **Intercept** for relapse: 1.704  **Intercept** for decade: -1.052  **Slope** for relapse: -0.258 | 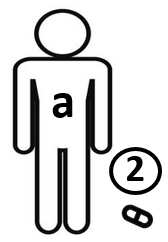  1991 – 2000 | Intercept: -1.519  (= -1.584-0.587+1.704  -1.052)  Slope: -0.155  (= 0.103-0.258) |

a=autologous; A=allogeneic

**SUPPLEMENTAL TABLE S1:** Additional characteristics of the included childhood cancer survivors (n=74)

|  | Total  (n=74) |
| --- | --- |
|  | n(%) |
| **Diagnosis according to ICCC3** |  |
| **Leukemia** | 51 (69%) |
| (Ia) Acute lymphoblastic leukemia | 30 (59) |
| (Ib) Acute myeloid leukemia | 12 (23) |
| (Ic) Chronic myeloproliferative syndrome | 4 (8) |
| (Id) Myelodysplastic syndrome | 5 (10) |
| **Lymphoma** | 11 (14%) |
| (IIa) Hodgkin lymophoma | 4 (36) |
| (IIb) Non-Hodgkin lymphoma | 5 (45) |
| (IIc) Burkitt lymphoma | 2 (18) |
| **Neuroblastoma** | 1 (1%) |
| **Other Tumors** | 11 (15%) |
| (IIIc) Embryonal brain tumor | 1 (9) |
| (V) Retinoblastoma | 1 (9) |
| (VIIIc) Ewing tumor | 5 (45) |
| (IXd) Other specified soft tissue  sarcoma | 3 (27) |
| (Xc) Malignant gonadal germ cell tumor | 1 (9) |
|  |  |
| **Source of transplant** |  |
| Cord blood | 5 (7) |
| Peripheral blood | 41 (55) |
| Bone marrow | 26 (35) |
| Unknown | 2 (3) |
|  |  |
| **Specific transplant characteristics in allogeneic population (n=50)** | |
| **Graft versus Host Disease** |  |
| No | 8 (16) |
| Yes | 42 (84) |
| Unknown | 1 (2) |
| Acute | 33 (79) |
| Chronic | 6 (14) |
| Skin | 5 (83) |
| Skin and other location | 1 (17) |

Abbreviations: ICCC-3, International Classification of Childhood Cancer, 3^rd^ edition

**SUPPLEMENTAL TABLE S2:** Results from linear mixed-effect multilevel regression model for **FEV1** z-scores, testing interaction with time since diagnosis for each risk factor separately and stratified by time since diagnosis (sensitivity analysis) in childhood cancer survivors treated with hematopoietic stem cell transplantation.

|  | **Whole period (max. 15 years since diagnosis)**  (407 tests, 74 CCSs) | | | | **First five years since diagnosis**  (138 tests, 50 CCSs) | | | |  |
| --- | --- | --- | --- | --- | --- | --- | --- | --- | --- |
|  | **Coefficient** | **P>\|z\|** | **95% Conf. Interval** | | **Coefficient** | **P>\|z\|** | **95% Conf. Interval** | |  |
| **Model without time interaction** |  |  |  |  |  |  |  |  |  |
| **Intercept** | 0.499 | 0.69 | -0.921 | 1.919 | 0.390 | 0.653 | -1.313 | 2.094 |  |
| **Gender** (ref. male) | -0.664 | **0.013** | -1.187 | -0.140 | -0.877 | **0.012** | -1.558 | -0.196 |  |
| **Type of HSCT** (ref. autologous) | 0.481 | 0.113 | -0.113 | 1.076 | 0.288 | 0.435 | -0.435 | 1.011 |  |
| **Radiotherapy to lung** (ref. no) | -1.306 | **0.001** | -2.055 | -0.558 | -1.198 | **0.006** | -2.047 | -0.349 |  |
| **Lung toxic chemotherapy** (ref. no) | -0.559 | 0.123 | -1.270 | 0.152 | -0.351 | 0.387 | -1.145 | 0.443 |  |
| **Relapse** (ref. = no) | 0.395 | 0.154 | -0.148 | 0.937 | -0.076 | 0.834 | -0.795 | 0.641 |  |
| **Decade of diagnosis** (ref. 1980-1990) |  |  |  |  |  |  |  |  |  |
| 1991 – 2000 | -0.667 | 0.183 | -1.647 | 0.314 | -0.732 | 0.242 | -1.957 | 0.493 |  |
| 2001 - 2010 | -0.197 | 0.678 | -1.131 | 0.736 | -0.155 | 0.795 | -1.318 | 1.009 |  |
| **Time since diagnosis** [decrease per year] | -0.061 | **<0.001** | -0.094 | -0.027 | -0.024 | 0.682 | -0.137 | 0.090 |  |
|  |  |  |  |  |  |  |  |  |  |
| **Interaction with time from diagnosis^1^** |  |  |  | |  |  |  | |  |
| **Gender** | 0.018 | 0.602 | -0.051 | 0.089 | -0.195 | 0.074 | -0.409 | 0.018 |  |
| **Type of HSCT** | 0.015 | 0.677 | -0.057 | 0.088 | 0.108 | 0.348 | -0.118 | 0.335 |  |
| **Radiotherapy** | 0.007 | 0.849 | -0.070 | 0.085 | -0.047 | 0.726 | -0.309 | 0.215 |  |
| **Lung toxic chemotherapy** | -0.009 | 0.793 | -0.077 | 0.059 | 0.159 | 0.159 | -0.380 | 0.062 |  |
| **Relapse** | 0.008 | 0.820 | -0.059 | 0.075 | -0.107 | 0.380 | -0.346 | 0.132 |  |
| **Decade of diagnosis** |  |  |  |  |  |  |  |  |  |
| 1991 – 2000 | -0.015 | 0.808 | -0.132 | 0.104 | 0.633 | **0.038** | 0.036 | 1.230 |  |
| 2001 - 2010 | 0.0004 | 0.994 | -1.044 | 1.984 | 0.561 | 0.056 | -0.014 | 1.137 |  |
|  |  |  |  |  |  |  |  |  |  |
| **Final Model** |  |  |  | |  |  |  | |  |
| **Intercept** | 0.499 | 0.69 | -0.921 | 1.919 | 1.406 | 0.154 | -0.529 | 3.341 |  |
| **Gender** (ref. male) | -0.664 | **0.013** | -1.187 | -0.140 | -0.919 | **0.007** | -1.584 | -0.255 |  |
| **Type of HSCT** (ref. autologous) | 0.481 | 0.113 | -0.113 | 1.076 | 0.230 | 0.521 | -0.473 | 0.934 |  |
| **Radiotherapy to lung** (ref. no) | -1.306 | **0.001** | -2.055 | -0.558 | -1.222 | **0.004** | -2.047 | -0.397 |  |
| **Lung toxic chemotherapy** (ref. no) | -0.559 | 0.123 | -1.270 | 0.152 | -0.349 | 0.375 | -1.120 | 0.422 |  |
| **Relapse** (ref. no) | 0.395 | 0.154 | -0.148 | 0.937 | -0.096 | 0.789 | -0.796 | 0.605 |  |
| **Decade of diagnosis** (ref. 1980-1990) |  |  |  |  |  |  |  |  |  |
| 1991 – 2000 | -0.667 | 0.183 | -1.647 | 0.314 | -1.897 | **0.022** | -3.517 | -0.277 |  |
| 2001 - 2010 | -0.197 | 0.678 | -1.131 | 0.736 | -1.098 | 0.056 | -2.597 | 0.401 |  |
| **Time since diagnosis** (cont. per year) | -0.061 | **<0.001** | -0.094 | -0.027 | -0.583 | **0.041** | -1.143 | 0.024 |  |
| **Interaction decade of diagnosis** |  |  |  |  |  |  |  |  |  |
| 1991 – 2000 | - | - | - | - | 0.633 | **0.038** | 0.036 | 1.230 |  |
| 2001 - 2010 | - | - | - | - | 0.561 | 0.056 | -0.014 | 1.137 |  |
|  |  |  |  |  |  |  |  |  |  |

^1^ Interaction with time assesses whether the exposure to each of the risk factors has a significant impact on the annual change in the FEV1 z-score (e.g. does the annual change differ between male and female CCSs, or CCSs exposed to radiotherapy or not)

**SUPPLEMENTAL TABLE S3:** Results from linear mixed-effect multilevel regression model for **FVC** z-scores, testing interaction with time since diagnosis for each risk factor separately and stratified by time since diagnosis (sensitivity analysis) in childhood cancer survivors treated with hematopoietic stem cell transplantation.

|  | **Whole period (max. 15 years since diagnosis)**  (395 tests, 73 CCSs) | | | | **First five years since diagnosis**  (126 tests, 46 CCSs) | | | |
| --- | --- | --- | --- | --- | --- | --- | --- | --- |
|  | **Coefficient** | **P>\|z\|** | **95% Conf. Interval** | | **Coefficient** | **P>\|z\|** | **95% Conf. Interval** | |
| **Model without time-interaction** |  |  |  |  |  |  |  |  |
| **Intercept** | 0.147 | 0.837 | -1.252 | 1.546 | 0.054 | 0.952 | -1.691 | 1.799 |
| **Gender** (ref. male) | -0.387 | 0.152 | -0.916 | 0.143 | -0.725 | **0.046** | -1.436 | -0.013 |
| **Type of HSCT** (ref. autologous) | 0.533 | 0.077 | -0.058 | 1.123 | 0.251 | 0.509 | -0.495 | 0.997 |
| **Radiotherapy to lung** (ref. no) | -1.473 | **<0.001** | -2.207 | -0.739 | -1.618 | **<0.001** | -2.479 | -0.756 |
| **Lung toxic chemotherapy** (ref. no) | -0.647 | 0.069 | -1.347 | 0.051 | -0.666 | 0.106 | -1.474 | 0.142 |
| **Relapse** (ref. = no) | 0.237 | 0.395 | -0.309 | 0.783 | 0.172 | 0.649 | -0.566 | 0.909 |
| **Decade of diagnosis** (ref. 1980-1990) |  |  |  |  |  |  |  |  |
| 1991 – 2000 | -0.312 | 0.531 | -1.287 | 0.663 | -0.096 | 0.882 | -1.373 | 1.181 |
| 2001 - 2010 | -0.083 | 0.861 | -1.008 | 0.843 | -0.198 | 0.746 | -1.005 | 1.402 |
| **Time since diagnosis** [decrease per year] | -0.058 | **0.003** | -0.097 | -0.019 | -0.016 | 0.806 | -0.142 | 0.110 |
|  |  |  |  |  |  |  |  |  |
| **Interaction with time from diagnosis^1^** |  |  |  | |  |  |  | |
| **Gender** | 0.040 | 0.327 | -0.040 | 0.121 | -0.076 | 0.548 | -0.324 | 0.172 |
| **Type of HSCT** | 0.007 | 0.869 | -0.076 | 0.089 | 0.062 | 0.627 | -0.189 | 0.313 |
| **Radiotherapy** | -0.006 | 0.888 | -0.094 | 0.081 | -0.132 | 0.346 | -0.408 | 0.143 |
| **Lung toxic chemotherapy** | 0.015 | 0.709 | -0.063 | 0.093 | -0.108 | 0.390 | -0.356 | 0.139 |
| **Relapse** | -0.021 | 0.602 | -0.098 | 0.057 | -0.066 | 0.624 | -0.328 | 0.197 |
| **Decade of diagnosis** |  |  |  |  |  |  |  |  |
| 1991 – 2000 | -0.017 | 0.808 | -0.155 | 0.121 | 0.795 | **0.010** | 0.193 | 1.397 |
| 2001 - 2010 | 0.019 | 0.770 | -0.112 | 0.151 | 0.740 | **0.009** | 0.181 | 1.299 |
|  |  |  |  |  |  |  |  |  |
| **Final Model** |  |  |  |  |  |  |  |  |
| **Intercept** | 0.147 | 0.837 | -1.252 | 1.546 | 1.571 | 0.125 | -0.435 | 3.579 |
| **Gender** (ref. male) | -0.387 | 0.152 | -0.916 | 0.143 | -0.799 | **0.023** | -1.489 | -0.109 |
| **Type of HSCT** (ref. autologous) | 0.533 | 0.077 | -0.058 | 1.123 | 0.167 | 0.648 | -0.552 | 0.887 |
| **Radiotherapy to lung** (ref. no) | -1.473 | **<0.001** | -2.207 | -0.739 | -1.615 | **<0.001** | -2.442 | -0.788 |
| **Lung toxic chemotherapy** (ref. no) | -0.647 | 0.069 | -1.347 | 0.051 | -0.659 | 0.096 | -1.436 | 0.117 |
| **Relapse** (ref. no) | 0.237 | 0.395 | -0.309 | 0.783 | 0.155 | 0.670 | -0.558 | 0.867 |
| **Decade of diagnosis** (ref. 1980-1990) |  |  |  |  |  |  |  |  |
| 1991 – 2000 | -0.312 | 0.531 | -1.287 | 0.663 | -1.811 | **0.050** | -3.624 | 0.003 |
| 2001 - 2010 | -0.083 | 0.861 | -1.008 | 0.843 | -1.308 | 0.112 | -2.921 | 0.303 |
| **Time since diagnosis** (continuous per year) | -0.058 | **0.003** | -0.097 | -0.019 | -0.731 | **0.008** | -1.272 | -0.189 |
| **Interaction decade of diagnosis** |  |  |  |  |  |  |  |  |
| 1991 – 2000 | - | - | - | - | 0.795 | **0.010** | 0.193 | 1.396 |
| 2001 - 2010 | - | - | - | - | 0.740 | **0.009** | 0.181 | 1.299 |
|  |  |  |  |  |  |  |  |  |

^1^ Interaction with time assesses whether the exposure to each of the risk factors has a significant impact on the annual change in the FEV1 z-score (e.g. does the annual change differ between male and female CCSs, or CCSs exposed to radiotherapy or not)

**SUPPLEMENTAL TABLE S4:** Results from linear mixed-effect multilevel regression model for **MMEF** z-scores, testing interaction with time since diagnosis for each risk factor separately and stratified by time since diagnosis (sensitivity analysis) in childhood cancer survivors treated with hematopoietic stem cell transplantation.

|  | **Whole period (max. 15 years since diagnosis)**  (268 tests, 61 CCSs) | | | | **First five years since diagnosis**  (96 tests, 39 CCSs) | | | |
| --- | --- | --- | --- | --- | --- | --- | --- | --- |
|  | **Coefficient** | **P>\|z\|** | **95% Conf. Interval** | | **Coefficient** | **P>\|z\|** | **95% Conf. Interval** | |
| **Model without time-interaction (Final model)** |  |  |  |  |  |  |  |  |
| **Intercept** | 1.340 | 0.146 | -0.469 | 3.149 | 0.148 | 0.890 | -1.957 | 2.255 |
| **Gender** (ref. male) | -0.887 | **0.008** | -1.538 | -0.237 | -1.107 | **0.008** | -1.921 | -0.294 |
| **Type of HSCT** (ref. autologous) | 0.402 | 0.328 | -0.404 | 1.208 | 0.812 | 0.085 | -0.111 | 1.737 |
| **Radiotherapy to lung** (ref. no) | -0.664 | 0.156 | -1.583 | 0.253 | -0.328 | 0.544 | -1.338 | 0.732 |
| **Lung toxic chemotherapy** (ref. no) | -0.494 | 0.275 | -1.383 | 0.394 | -0.138 | 0.779 | -1.104 | 0.828 |
| **Relapse** (ref. = no) | 0.374 | 0.252 | -0.266 | 1.016 | 0.098 | 0.814 | -0.727 | 0.925 |
| **Decade of diagnosis** (ref. 1980-1990) |  |  |  |  |  |  |  |  |
| 1991 – 2000 | -1.142 | 0.059 | -2.327 | 0.042 | -0.845 | 0.252 | -2.290 | 0.600 |
| 2001 - 2010 | -0.982 | 0.106 | -2.172 | 0.208 | -1.033 | 0.182 | -2.553 | 0.485 |
| **Time since diagnosis** [decrease per year] | -0.003 | 0.918 | -0.058 | 0.052 | 0.198 | **0.026** | 0.024 | 0.373 |
|  |  |  |  |  |  |  |  |  |
| **Interaction with time from diagnosis^1^** | **Coefficient** | **P>\|z\|** | **95% Conf. Interval** | | **Coefficient** | **P>\|z\|** | **95% Conf. Interval** | |
| **Gender** | -0.024 | 0.690 | -0.141 | 0.093 | -0.215 | 0.207 | -0.549 | 0.119 |
| **Type of HSCT*** | -0.007 | 0.915 | -0.141 | 0.126 | 0.130 | 0.487 | -0.237 | 0.497 |
| **Radiotherapy** | -0.017 | 0.771 | -0.135 | 0.100 | -0.048 | 0.808 | -0.439 | 0.342 |
| **Lung toxic chemotherapy** | -0.022 | 0.694 | -0.132 | 0.088 | -0.002 | 0.988 | -0.361 | 0.355 |
| **Relapse** | -0.014 | 0.812 | -0.125 | 0.097 | -0.271 | 0.155 | -0.644 | 0.102 |
| **Decade of diagnosis*** |  |  |  |  |  |  |  |  |
| 1991 – 2000 | -0.122 | 0.229 | -0.323 | 0.077 | -0.042 | 0.917 | -0.825 | 0.742 |
| 2001 - 2010 | -0.026 | 0.787 | -0.212 | 0.161 | 0.101 | 0.787 | -0.629 | 0.830 |
|  |  |  |  |  |  |  |  |  |

^1^ Interaction with time assesses whether the exposure to each of the risk factors has a significant impact on the annual change in the FVC z-score (e.g. does the annual change differ between male and female CCSs, or CCSs exposed to radiotherapy or not)

* autocorrelation of residuals omitted because convergence not achievable

**SUPPLEMENTAL TABLE S5:** Results from linear mixed-effect multilevel regression model for **TLC** z-scores, testing interaction with time since diagnosis for each risk factor separately and stratified by time since diagnosis (sensitivity analysis) in childhood cancer survivors treated with hematopoietic stem cell transplantation.

|  | **Whole period (max. 15 years since diagnosis)**  (390 tests, 74 CCSs) | | | | **First five years since diagnosis**  (133 tests, 48 CCSs) | | | |
| --- | --- | --- | --- | --- | --- | --- | --- | --- |
|  | **Coefficient** | **P>\|z\|** | **95% Conf. Interval** | | **Coefficient** | **P>\|z\|** | **95% Conf. Interval** | |
| **Model without time-interaction*** |  |  |  |  |  |  |  |  |
| **Intercept** | -1.245 | 0.342 | -3.816 | 1.326 | -2.102 | 0.137 | -4.874 | 0.670 |
| **Gender** (ref. male) | 0.628 | 0.194 | -0.320 | 1.576 | 0.332 | 0.565 | -0.799 | 1.464 |
| **Type of HSCT** (ref. autologous) | 0.325 | 0.550 | -0.738 | 1.388 | 0.025 | 0.967 | -1.162 | 1.211 |
| **Radiotherapy to lung** (ref. no) | -0.788 | 0.246 | -2.120 | 0.543 | -1.061 | 0.124 | -2.411 | 0.289 |
| **Lung toxic chemotherapy** (ref. no) | -0.375 | 0.558 | -1.632 | 0.881 | 0.357 | 0.586 | -0.929 | 1.644 |
| **Relapse** (ref. = no) | 0.135 | 0.787 | -0.845 | 1.116 | 1.031 | 0.113 | -0.245 | 2.307 |
| **Decade of diagnosis** (ref. 1980-1990) |  |  |  |  |  |  |  |  |
| 1991 – 2000 | -1.104 | 0.220 | -2.869 | 0.660 | 0.714 | 0.476 | -1.249 | 2.676 |
| 2001 - 2010 | -0.280 | 0.743 | -1.955 | 1.394 | 0.479 | 0.601 | -1.320 | 2.279 |
| **Time since diagnosis** [decrease per year] | 0.047 | 0.274 | -0.037 | 0.130 | -0.080 | 0.533 | -0.332 | 0.172 |
|  |  |  |  |  |  |  |  |  |
| **Interaction with time from diagnosis^1^** |  |  |  | |  |  |  | |
| **Gender** | 0.016 | 0.857 | -0.159 | 0.192 | -0.078 | 0.758 | -0.573 | 0.417 |
| **Type of HSCT** | 0.216 | **0.007** | 0.059 | 0.372 | 0.044 | 0.862 | -0.452 | 0.539 |
| **Radiotherapy** | -0.034 | 0.733 | -0.227 | 0.159 | -0.089 | 0.756 | -0.652 | 0.474 |
| **Lung toxic chemotherapy** | -0.060 | 0.472 | -0.225 | 0.104 | -0.503 | **0.039** | -0.981 | -0.026 |
| **Relapse*** | -0.308 | **<0.001** | -0.457 | -0.157 | -0.239 | 0.360 | -0.752 | -0.273 |
| **Decade of diagnosis** |  |  |  |  |  |  |  |  |
| 1991 – 2000 | 0.003 | 0.979 | -0.238 | 0.245 | 0.912 | 0.135 | -0.284 | 2.109 |
| 2001 - 2010 | 0.163 | 0.170 | -0.069 | 0.395 | 0.726 | 0.212 | -0.415 | 1.868 |
|  |  |  |  |  |  |  |  |  |
| **Final Model** |  |  |  | |  |  |  | |
| **Intercept** | -1.584 | 0.251 | -4.293 | 1.124 | -2.308 | 0.092 | -4.994 | 0.377 |
| **Gender** (ref. male) | 0.729 | 0.134 | -0.224 | 1.682 | 0.239 | 0.667 | -0.851 | 1.331 |
| **Type of HSCT** (ref. autologous) | -0.510 | 0.479 | -1.923 | 0.903 | -0.060 | 0.918 | -1.208 | 1.087 |
| **Radiotherapy to lung** (ref. no) | -0.717 | 0.292 | -2.051 | 0.616 | -1.135 | 0.088 | -2.439 | 0.168 |
| **Lung toxic chemotherapy** (ref. no) | -0.587 | 0.364 | -1.855 | 0.681 | 1.278 | 0.101 | -0.247 | 2.804 |
| **Relapse** (ref. no) | 1.704 | **0.015** | 0.333 | 3.075 | 0.877 | 0.165 | -0.361 | 2.115 |
| **Decade of diagnosis** (ref. 1980-1990) |  |  |  |  |  |  |  |  |
| 1991 – 2000 | -1.052 | 0.242 | -2.814 | 0.711 | 0.642 | 0.506 | -1.251 | 2.536 |
| 2001 - 2010 | -0.204 | 0.811 | -1.874 | 1.465 | 0.507 | 0.566 | -1.226 | 2.241 |
| **Time since diagnosis** (continuous per year) | 0.103 | 0.236 | -0.067 | 0.272 | 0.127 | 0.434 | -0.191 | 0.445 |
| **Interaction Type of HSCT** (ref. autologous) | 0.123 | 0.136 | -0.038 | 0.284 | - | - | - | - |
| **Interaction relapse** (ref. no) | -0.258 | **0.001** | -0.414 | -0.103 | - | - | - | - |
| **Interaction chemotherapy** (ref. no) | - | - | - | - | -0.503 | **0.039** | -0.981 | -0.026 |
|  |  |  |  |  |  |  |  |  |

^1^ Interaction with time assesses whether the exposure to each of the risk factors has a significant impact on the annual change in the FEV1 z-score (e.g. does the annual change differ between male and female CCSs, or CCSs exposed to radiotherapy or not)

* autocorrelation of residuals omitted because convergence not achievable

**SUPPLEMENTAL TABLE S6:** Results from linear mixed-effect multilevel regression model for **RV** z-scores, testing interaction with time since diagnosis for each risk factor separately and stratified by time since diagnosis (sensitivity analysis) in childhood cancer survivors treated with hematopoietic stem cell transplantation.

|  | **Whole period (max. 15 years since diagnosis)**  (382 tests, 74 CCSs) | | | | **First five years since diagnosis**  (130 tests, 47 CCSs) | | | |
| --- | --- | --- | --- | --- | --- | --- | --- | --- |
|  | **Coefficient** | **P>\|z\|** | **95% Conf. Interval** | | **Coefficient** | **P>\|z\|** | **95% Conf. Interval** | |
| **Model without time-interaction** |  |  |  |  |  |  |  |  |
| **Intercept** | 0.481 | 0.631 | -1.478 | 2.439 | -0.266 | 0.848 | -2.987 | 2.454 |
| **Gender** (ref. male) | -0.072 | 0.836 | -0.752 | 0.609 | -0.038 | 0.947 | -1.149 | 1.074 |
| **Type of HSCT** (ref. autologous) | -0.084 | 0.831 | -0.849 | 0.682 | -0.703 | 0.235 | -1.864 | 0.457 |
| **Radiotherapy to lung** (ref. no) | 0.562 | 0.259 | -0.413 | 1.537 | 0.656 | 0.329 | -0.661 | 1.974 |
| **Lung toxic chemotherapy** (ref. no) | -0.192 | 0.676 | -1.088 | 0.705 | 0.404 | 0.532 | -0.864 | 1.671 |
| **Relapse** (ref. = no) | -0.330 | 0.348 | -1.019 | 0.359 | 0.148 | 0.814 | -1.092 | 1.390 |
| **Decade of diagnosis** (ref. 1980-1990) |  |  |  |  |  |  |  |  |
| 1991 – 2000 | -0.790 | 0.224 | -2.063 | 0.483 | 1.049 | 0.286 | -0.879 | 2.978 |
| 2001 – 2010 | -0.160 | 0.798 | -1.387 | 1.067 | 0.359 | 0.689 | -1.401 | 2.121 |
| **Time since diagnosis** [decrease per year] | -0.016 | 0.750 | -0.112 | 0.081 | -0.144 | 0.292 | -0.411 | 0.123 |
|  |  |  |  |  |  |  |  |  |
| **Interaction with time from diagnosis^1^** |  |  |  | |  |  |  | |
| **Gender** | 0.054 | 0.591 | -0.144 | 0.252 | -0.249 | 0.339 | -0.759 | 0.261 |
| **Type of HSCT** | 0.166 | 0.077 | -0.018 | 0.349 | 0.403 | 0.115 | -0.097 | 0.905 |
| **Radiotherapy** | -0.071 | 0.522 | -0.289 | 0.147 | -0.432 | 0.176 | -1.056 | 0.193 |
| **Lung toxic chemotherapy** | -0.036 | 0.718 | -0.229 | 0.158 | -0.126 | 0.637 | -0.651 | 0.398 |
| **Relapse** | -0.231 | **0.010** | -0.405 | -0.056 | 0.222 | 0.414 | -0.311 | 0.754 |
| **Decade of diagnosis** |  |  |  |  |  |  |  |  |
| 1991 – 2000 | -0.149 | 0.316 | -0.440 | 0.142 | 0.459 | 0.479 | -0.814 | 1.734 |
| 2001 - 2010 | 0.054 | 0.708 | -0.226 | 0.333 | 0.352 | 0.569 | -0.857 | 1.560 |
|  |  |  |  |  |  |  |  |  |
| **Final Model** |  |  |  | |  |  |  | |
| **Intercept** | -0.309 | 0.764 | -2.326 | 1.707 | -0.266 | 0.848 | -2.987 | 2.454 |
| **Gender** (ref. male) | 0.036 | 0.918 | -0.650 | 0.722 | -0.038 | 0.947 | -1.149 | 1.074 |
| **Type of HSCT** (ref. autologous) | -0.155 | 0.692 | -0.923 | 0.612 | -0.703 | 0.235 | -1.864 | 0.457 |
| **Radiotherapy to lung** (ref. no) | 0.663 | 0.181 | -0.307 | 1.634 | 0.656 | 0.329 | -0.661 | 1.974 |
| **Lung toxic chemotherapy** (ref. no) | -0.298 | 0.518 | -1.202 | 0.606 | 0.404 | 0.532 | -0.864 | 1.671 |
| **Relapse** (ref. = no) | 1.085 | 0.100 | -0.208 | 2.378 | 0.148 | 0.814 | -1.092 | 1.390 |
| **Decade of diagnosis** (ref. 1980-1990) | -0.785 | 0.226 | -2.055 | 0.485 |  |  |  |  |
| 1991 – 2000 | -0.127 | 0.838 | -1.346 | 1.902 | 1.049 | 0.286 | -0.879 | 2.978 |
| 2001 - 2010 |  |  |  |  | 0.359 | 0.689 | -1.401 | 2.121 |
| **Time since diagnosis** (continuous per year) | 0.108 | 0.095 | -0.019 | 0.234 | -0.144 | 0.292 | -0.411 | 0.123 |
| **Interaction with relapse** (ref. no) | -0.231 | **0.010** | -0.405 | -0.055 | - | - | - | - |
|  |  |  |  |  |  |  |  |  |

^1^ Interaction with time assesses whether the exposure to each of the risk factors has a significant impact on the annual change in the FEV1 z-score (e.g. does the annual change differ between male and female CCSs, or CCSs exposed to radiotherapy or not)

**SUPPLEMENTAL TABLE S7:** Results from linear mixed-effect multilevel regression model for **DLCO** z-scores, testing interaction with time since diagnosis for each risk factor separately and stratified by time since diagnosis (sensitivity analysis) in childhood cancer survivors treated with hematopoietic stem cell transplantation.

|  | **Whole period (max. 15 years since diagnosis)**  (185 tests, 46 CCSs) | | | | **First five years since diagnosis**  (50 tests, 23 CCSs) | | | |
| --- | --- | --- | --- | --- | --- | --- | --- | --- |
|  | **Coefficient** | **P>\|z\|** | **95% Conf. Interval** | | **Coefficient** | **P>\|z\|** | **95% Conf. Interval** | |
| **Model without time-interaction** |  |  |  |  |  |  |  |  |
| **Intercept** | 1.948 | 0.192 | -0.977 | 4.872 | 10.576 | **<0.001** | 6.532 | 14.621 |
| **Gender** (ref. male) | -0.514 | 0.341 | -1.575 | 0.546 | -3.569 | **<0.001** | -5.013 | -2.127 |
| **Type of HSCT** (ref. autologous) | 0.498 | 0.381 | -0.616 | 1.613 | -0.448 | 0.436 | -1.577 | 0.681 |
| **Radiotherapy to lung** (ref. no) | -1.279 | 0.093 | -2.773 | 0.213 | -4.159 | **<0.001** | -5.440 | -2.879 |
| **Lung toxic chemotherapy** (ref. no) | -0.707 | 0.296 | -2.033 | 0.619 | -0.694 | 0.208 | -1.776 | 0.387 |
| **Relapse** (ref. = no) | 0.138 | 0.809 | -0.986 | 1.263 | -2.026 | **0.010** | -3.563 | -0.490 |
| **Decade of diagnosis** (ref. 1980-1990) |  |  |  |  |  |  |  |  |
| 1991 – 2000 | -2.465 | **0.004** | -4.151 | -0.780 | -7.013 | **<0.001** | -9.228 | -4.799 |
| 2001 - 2010 | -2.447 | **0.004** | -4.111 | -0.784 | -8.093 | **<0.001** | -10.294 | -5.893 |
| **Time since diagnosis** [decrease per year] | 0.015 | 0.748 | -0.079 | 0.111 | 0.762 | 0.050 | 0.0009 | 1.523 |
|  |  |  |  |  |  |  |  |  |
| **Interaction with time from diagnosis^1^** |  |  |  | |  |  |  | |
| **Gender** | 0.183 | 0.063 | -0.010 | 0.376 | -1.331 | 0.066 | -2.751 | 0.089 |
| **Type of HSCT** | 0.059 | 0.558 | -0.138 | 0.256 | 0.444 | 0.661 | -1.268 | 2.156 |
| **Radiotherapy** | -0.025 | 0.871 | -0.325 | 0.275 | -0.026 | 0.963 | -1.141 | 1.088 |
| **Lung toxic chemotherapy** | -0.050 | 0.622 | -0.251 | 0.149 | -0.644 | 0.150 | -1.521 | 0.232 |
| **Relapse** | 0.051 | 0.627 | -0.156 | 0.259 | 1.491 | 0.053 | -0.021 | 3.004 |
| **Decade of diagnosis** |  |  |  |  |  |  |  |  |
| 1991 – 2000 | -0.128 | 0.302 | -0.371 | 0.115 | -2.800 | 0.085 | -5.986 | 0.385 |
| 2001 - 2010 | 0.012 | 0.885 | -0.249 | 0.289 | -3.285 | **0.040** | -6.417 | -0.154 |
|  |  |  |  |  |  |  |  |  |
| **Final Model** |  |  |  |  |  |  |  | |
| **Intercept** | 1.948 | 0.192 | -0.977 | 4.872 | 4.821 | 0.159 | -1.880 | 11.521 |
| **Gender** (ref. male) | -0.514 | 0.341 | -1.575 | 0.546 | -3.395 | **<0.001** | -4.805 | -1.984 |
| **Type of HSCT** (ref. autologous) | 0.498 | 0.381 | -0.616 | 1.613 | -0.487 | 0.386 | -1.588 | 0.614 |
| **Radiotherapy to lung** (ref. no) | -1.279 | 0.093 | -2.773 | 0.213 | -4.009 | **<0.001** | -5.261 | -2.757 |
| **Lung toxic chemotherapy** (ref. no) | -0.707 | 0.296 | -2.033 | 0.619 | -0.514 | 0.338 | -1.564 | 0.536 |
| **Relapse** (ref. = no) | 0.138 | 0.809 | -0.986 | 1.263 | -1.759 | **0.021** | -3.255 | -0.264 |
| **Decade of diagnosis** (ref. 1980-1990) |  |  |  |  |  |  |  |  |
| 1991 – 2000 | -2.465 | **0.004** | -4.151 | -0.780 | -2.016 | 0.534 | -8.370 | 4.337 |
| 2001 - 2010 | -2.447 | **0.004** | -4.111 | -0.784 | -1.785 | 0.573 | -7.991 | 4.422 |
| **Time since diagnosis** (continuous per year) | 0.015 | 0.748 | -0.079 | 0.111 | 3.659 | **0.016** | 0.685 | 6.634 |
| **Interaction with Decade** (ref. no) |  |  |  |  |  |  |  |  |
| 1991 – 2000 | - | - | - | - | -2.801 | 0.085 | -5.986 | 0.386 |
| 2001 - 2010 | - | - | - | - | -3.285 | **0.040** | -6.417 | -0.154 |
|  |  |  |  |  |  |  |  |  |

^1^ Interaction with time assesses whether the exposure to each of the risk factors has a significant impact on the annual change in the FEV1 z-score (e.g. does the annual change differ between male and female CCSs, or CCSs exposed to radiotherapy or not)

**SUPPLEMENTAL TABLE S8:** Characteristics of childhood cancer survivors with available baseline pulmonary function testing

| **FEV1** | | |
| --- | --- | --- |
|  | **N=24 at baseline** | **N=14 at >5 years** |
| **Gender**, male | 12 (50%) | 9 (64%) |
| **Ethnicity**, white | 24 (100%) | 13 (93%) |
| **Age at diagnosis** [years] | 9.1 (5.6 – 13.6) | 8.5 (5.6 – 9.7) |
| **Diagnosis**  Leukemia  Lymphoma  Other | 15 (62%)  3 (13%)  6 (25%) | 9 (64%)  2 (14%)  3 (22%) |
| **Era of diagnosis**  1980 – 1990  1991 – 2000  2001 - 2010 | 4 (17%)  6 (25%)  14 (58%) | 2 (14%)  4 (29%)  8 (57%) |
| **Relapse** | 17 (71%) | 10 (71%) |
| **FVC** |  |  |
|  | **N=23 at baseline** | **N=15 at >5 years** |
| **Gender**, male | 11 (48%) | 10 (67%) |
| **Ethnicity**, white | 23 (100%) | 14 (93%) |
| **Age at diagnosis** [years] | 9.1 (5.6 – 23.6) | 8.7 (5.6 – 12.5)) |
| **Diagnosis**  Leukemia  Lymphoma  Other | 14 (61%)  4 (17%)  5 (22%) | 10 (67%)  2 (13%)  3 (20%) |
| **Era of diagnosis**  1980 – 1990  1991 – 2000  2001 - 2010 | 3 (13%)  6 (26%)  14 (61%) | 2 (13%)  4 (27%)  9 (60%) |
| **Relapse** | 16 (69%) | 10 (67%) |


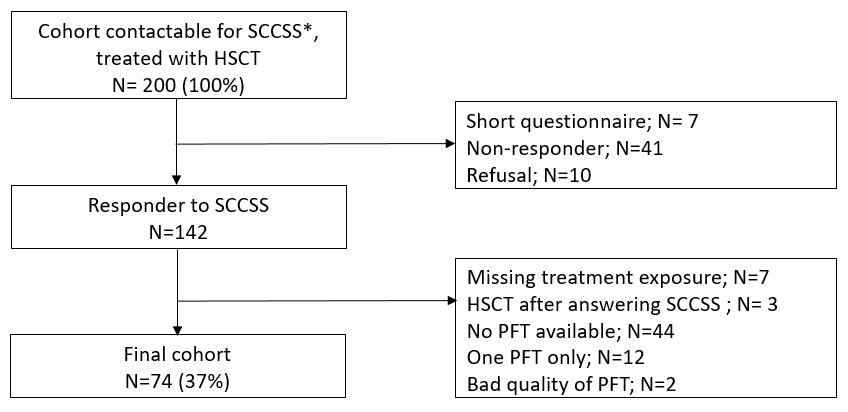


**SUPPLEMENTAL FIGURE S1:** Population tree of transplanted childhood cancer survivors eligible for this study.

*Contactable for SCCSS: diagnosed 1976-2010, 5-year survivor, alive, and address available

CCS, childhood cancer survivor; HSCT, hematopoietic stem cell transplantation; SCCSS, Swiss Childhood Cancer Survivor Study; PFT, pulmonary function test; Key questionnaire did not include questions on pulmonary health

**S2 A**
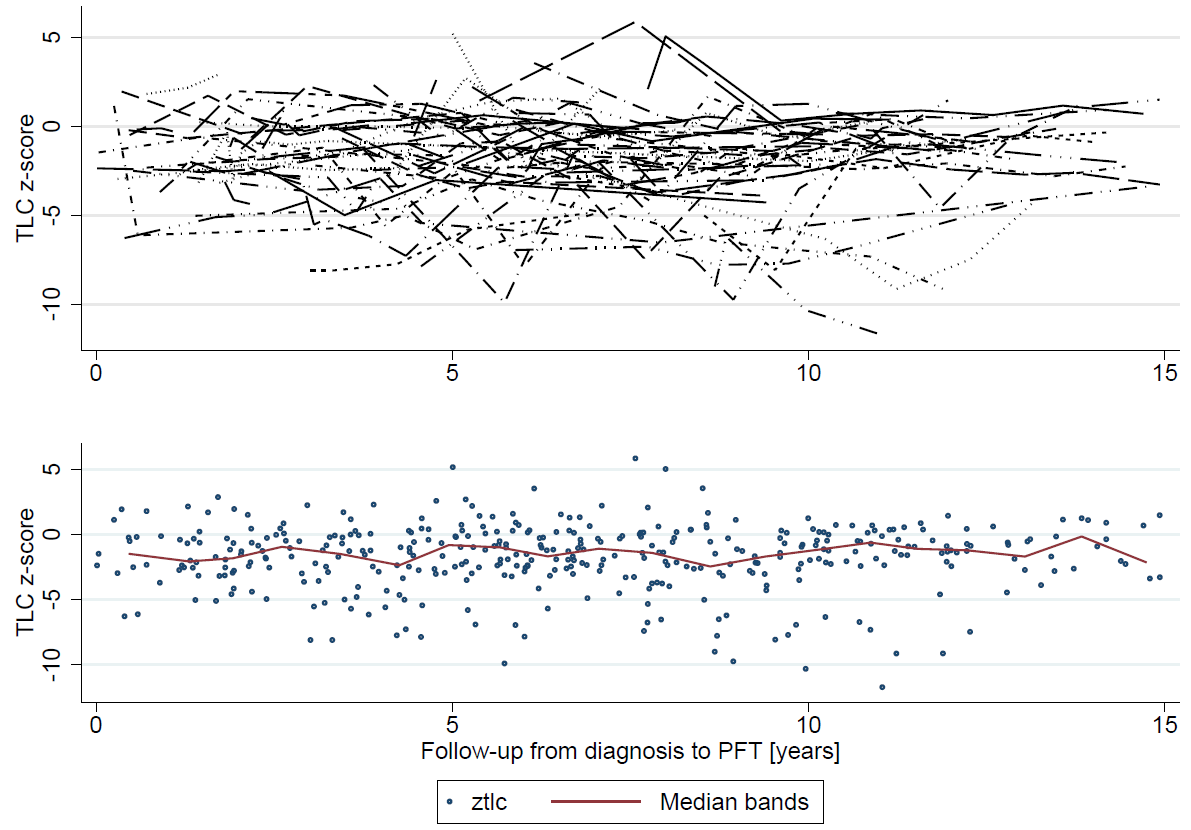


**S2 B**
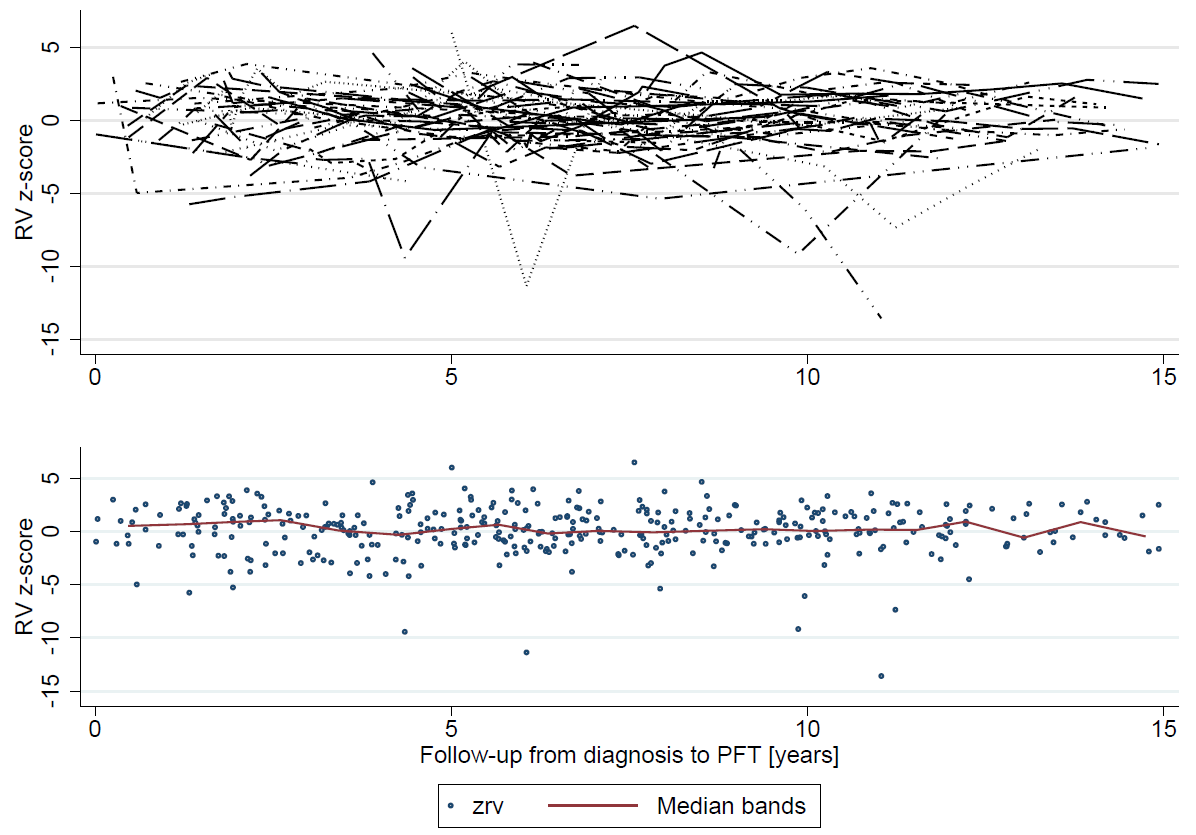


**S2 C**
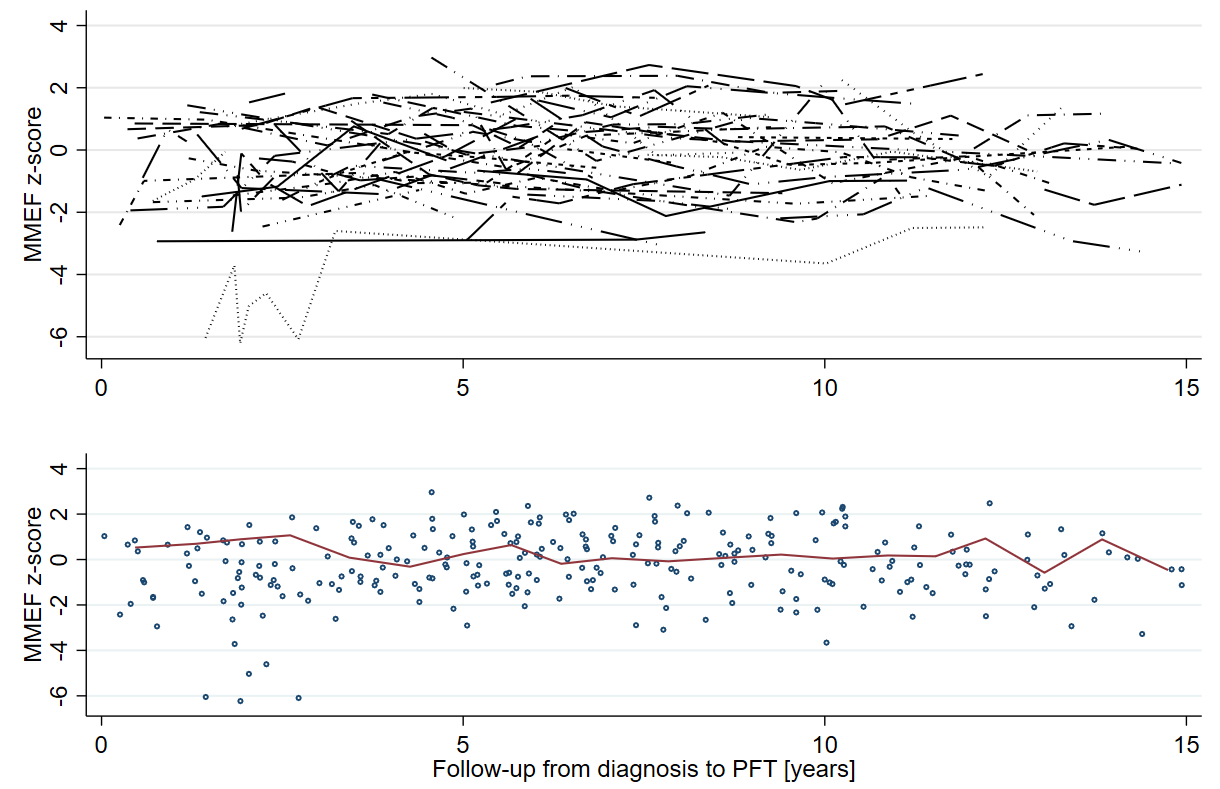


**SUPPLEMENTAL FIGURE S2:** Longitudinal trajectories of A) TLC z-score, B) RV z-score, and C) MMEF z-score over time, upper part showing the trajectory of each patient, lower part showing the median of all observations

**3A**
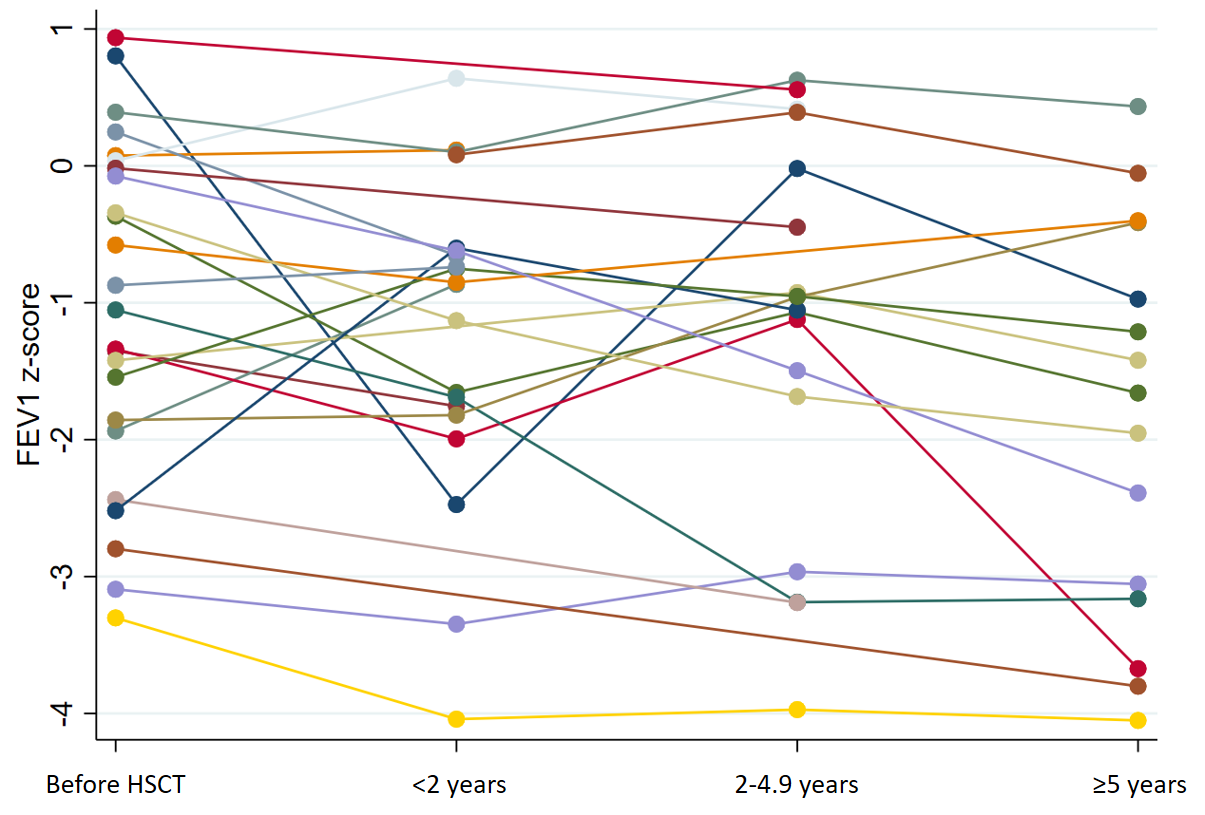


**3B**
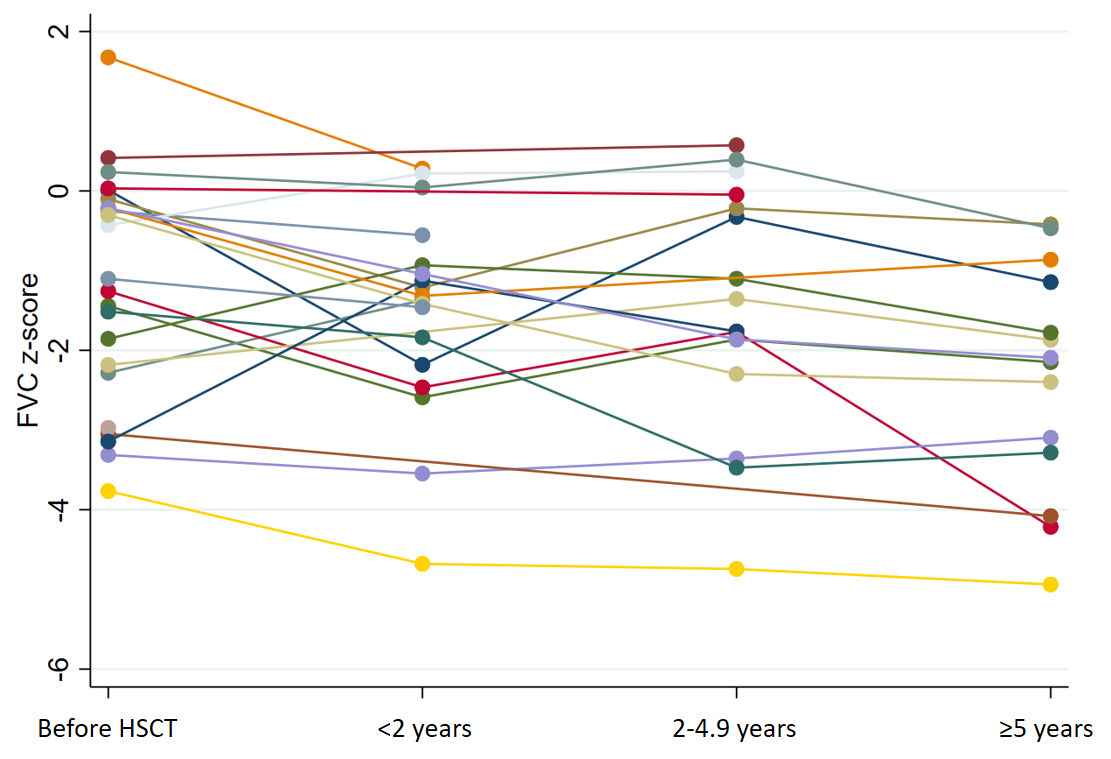


**SUPPLEMENTAL FIGURE S3:** Longitudinal trajectories of A) FEV1 z-score and B) FVC z-score in childhood cancer survivors with baseline testing before HSTC.
